# Machine Learning-Assisted Short-Wave InfraRed (SWIR) Techniques for Biomedical Applications: Towards Personalized Medicine

**DOI:** 10.3390/jpm14010033

**Published:** 2023-12-26

**Authors:** Mohammadhossein Salimi, Majid Roshanfar, Nima Tabatabaei, Bobak Mosadegh

**Affiliations:** 1 Department of Mechanical Engineering, York University, Toronto, ON M3J 1P3, Canada; mhsalimi@yorku.ca; 2 Department of Mechanical Engineering, Concordia University, Montreal, QC H3G 1M8, Canada; m_roshan@encs.concordia.ca; 3 Dalio Institute of Cardiovascular Imaging, Department of Radiology, Weill Cornell Medicine, New York, NY 10021, USA

**Keywords:** personalized medicine, short-wave infrared (SWIR) techniques, machine learning, deep learning, biomedical optics, individualized bioinstruments

## Abstract

Personalized medicine transforms healthcare by adapting interventions to individuals’ unique genetic, molecular, and clinical profiles. To maximize diagnostic and/or therapeutic efficacy, personalized medicine requires advanced imaging devices and sensors for accurate assessment and monitoring of individual patient conditions or responses to therapeutics. In the field of biomedical optics, short-wave infrared (SWIR) techniques offer an array of capabilities that hold promise to significantly enhance diagnostics, imaging, and therapeutic interventions. SWIR techniques provide in vivo information, which was previously inaccessible, by making use of its capacity to penetrate biological tissues with reduced attenuation and enable researchers and clinicians to delve deeper into anatomical structures, physiological processes, and molecular interactions. Combining SWIR techniques with machine learning (ML), which is a powerful tool for analyzing information, holds the potential to provide unprecedented accuracy for disease detection, precision in treatment guidance, and correlations of complex biological features, opening the way for the data-driven personalized medicine field. Despite numerous biomedical demonstrations that utilize cutting-edge SWIR techniques, the clinical potential of this approach has remained significantly underexplored. This paper demonstrates how the synergy between SWIR imaging and ML is reshaping biomedical research and clinical applications. As the paper showcases the growing significance of SWIR imaging techniques that are empowered by ML, it calls for continued collaboration between researchers, engineers, and clinicians to boost the translation of this technology into clinics, ultimately bridging the gap between cutting-edge technology and its potential for personalized medicine.

## 1. Introduction

In recent years, healthcare has undergone a profound transformation, driven by the emerging field of personalized medicine and the growing emphasis on individualized healthcare solutions [1,2,3]. Personalized medicine represents a departure from traditional, uniform healthcare approaches, offering a new frontier where technology adapts to the unique characteristics of individual health profiles. This shift in the medical paradigm is characterized by a fundamental recognition of the unique physiological and genetic makeup of each patient, requiring personalized medical interventions that go beyond the one-size-fits-all approach of the past. This has led to more effective treatments with fewer side effects, improving patient outcomes and overall quality of care. However, the future of personalized medicine holds even greater potential for revolutionizing healthcare. As our understanding of the human genome deepens and biomedical instruments continue to expand, we can anticipate the development of increasingly effective remedies. Central to this revolution is the integration of advanced technologies that can provide intricate insights into the inner workings of the human body.

The concept of “Individualized Bioinstruments” envisions the creation of medical devices and tools that are custom-tailored to the unique needs and conditions of each patient, and facilities a data-driven personalized medicine approach. Personalized medicine benefits significantly from individualized bioinstrumentation, as it furnishes crucial tools and technologies to facilitate customized healthcare solutions in different ways, including: precise diagnosis, personalized treatment plans, monitoring and adaptation, early detection, targeted therapies, optimization of drug regimens, and data-driven decision-making. Key requirements for suitable bioinstruments in this comprehensive context encompass but are not limited to: high-resolution imaging, interoperability, real-time monitoring, cost-effectiveness, privacy and security, scalability, customization, data integration, and data analytics. Hence, the imaging and sensory devices that satisfy these requirements are of utmost importance. Optical bioinstruments, with their focus on user safety, compact size, cost-effectiveness, and their intrinsic sensitivity to disease-induced changes in the structure and environment of biomolecules and biostructures, offer the promise for extracting patient information to enable tailored treatments.

Due to this transformative landscape, short-wave infrared (SWIR; 1000–1700 nm) techniques have emerged as a powerful and versatile optical tool for various biomedical applications that leverage the unique properties of SWIR light to penetrate biological tissues and provide high-resolution data about their composition and function, in vivo. Additionally, the SWIR range offers distinguishable light absorption spectra for key constituents of biological tissues, such as water, collagen, and lipids, making certain that SWIR techniques are sensitive and specific to both structural and molecular disease-induced anomalies [4]. This technology enables the visualization of specific biomarkers, molecular structures, and physiological dynamics within the body, providing a wealth of patient-specific data. SWIR techniques have great potential to contribute significantly to the development of individualized biomedical tools that can adapt to the distinct needs of each patient, offering both deep and high-resolution insights, which facilitate precise diagnostics and treatments in a patient-centric healthcare landscape.

**Figure 1:**
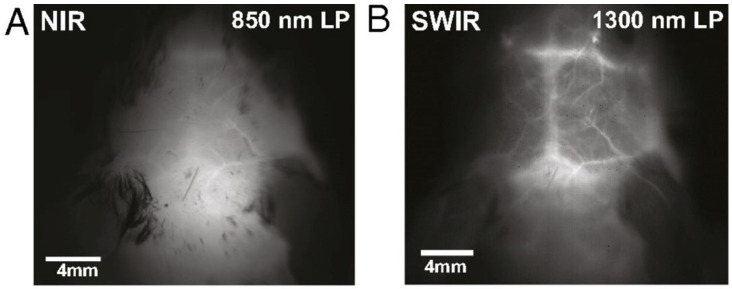
SWIR vs. NIR. Fluorescent images taken from a mouse head with a long pass (LP) filter in the (**A**) NIR range, and (**B**) SWIR range. Higher contrast with more details from vasculature can be seen in the SWIR image. Figure adapted with permission from [8].

Traditionally, the visible spectrum (400–700 nm) has been employed for imaging tissue in vivo, but its effectiveness is curtailed by the high scattering and absorption of visible light resulting in blurred images and limited penetration depth. To address these limitations for in vivo imaging, taking into account the inverse relationship between Rayleigh scattering and the wavelength of light, imaging at longer wavelengths has proven advantageous in terms of extending the imaging depth [5]. While these advantages are offered by both near infrared (NIR) (700–1000 nm) and SWIR (1000–1700 nm) ranges, the eventual imaging performance is better at the SWIR range when imaging bulky tissues. This advantage occurs in media where light scattering dominates light absorption, as in the case with bulky tissue. Therefore, there is an enhancement of image resolution since the reduction of light scattering at the longer SWIR wavelengths outweighs the enhancements offered by the smaller focal spot size (i.e., Airy disk size) at the shorter NIR wavelengths [5,6,7]. Figure 1 highlights the differences between captured in vivo images of mouse brain vasculature in NIR and SWIR. In addition to image resolution, utilizing longer SWIR wavelengths offer less autofluorescence and less absorption of light by the dominant constituents of biological tissues such as blood, fat, and melanin, which eventually leads to larger light penetration/imaging depths in biological tissues (~6 mm).

From a clinical perspective, the existing biomedical imaging systems (i.e., MRI, X-ray, and ultrasound) grapple with inherent limitations that impact their comprehensive utility in clinical environments. Magnetic resonance imaging (MRI) excels in providing detailed soft tissue images but faces challenges related to limited spatial resolution and prolonged scan times. X-ray imaging, while widely used for its ability to penetrate tissues and visualize bones, is constrained by its ionizing radiation and/or need for contrast agents, limiting its frequent application. Ultrasound, although valuable for real-time imaging and lack of ionizing radiation, encounters challenges with providing detailed anatomical information. In contrast, SWIR imaging emerges as a transformative solution, leveraging its unique ability to provide real-time high-resolution images using a safe frequency range for the radiation of light.

Recent technological advancements have made it possible to explore imaging beyond visible range with the introduction of commercially available SWIR cameras. This progress is attributed to improved fabrication of Indium Gallium Arsenide (InGaAs)-based sensors, resulting in relatively cost-effective SWIR cameras with heightened sensitivity and resolution. It should be noted that previously, SWIR cameras were primarily restricted for military use due to the International Traffic in Arms Regulations (ITAR). During this period, non-military applications only had access to line scan SWIR cameras, which were used in medical sensing and imaging devices utilizing spectrometers. Today, however, SWIR cameras are available, albeit with some restrictions, to researchers and the general public, and as a result, the applications of SWIR imaging are expanding in the biomedical field, especially in clinical settings [4,8,9,10,11,12,13,14,15]. The clinical SWIR images and data, however, are inherently multifactorial and non-linear, which makes their interpretation highly complex [16]. Consequently, the need for real-time and accurate processing methods for SWIR clinical results are increasingly imperative [17]. Machine learning (ML)—as a disruptive technology—holds the potential to revolutionize the biomedical sector by providing efficient solutions for processing of the multifactorial SWIR clinical data [18].

ML encompasses models and algorithms that iteratively enhance their performance through a learning process by interpreting relationships found in large amounts of data [19]. In the context of personalized medicine, ML algorithms can sift through vast and diverse patient data, including diagnostic imaging, medical histories, and genetic profiles, to identify intricate patterns, associations, and correlations that may be beyond the human capacity to discern. This analytical tool has great potential to empower healthcare professionals to precisely predict disease risks, recommend personalized treatment regimens, and even anticipate adverse events, all tailored to an individual’s unique biological makeup.

Particularly, when combined with ML techniques, SWIR imaging has the potential to provide highly detailed and patient-specific information about tissues and disease states. This technique can enable the creation of bioinstruments that are tailored to individual patients’ needs and conditions. For example, SWIR imaging could be used to analyze specific biomarkers or tissue characteristics in a patient’s body, and ML algorithms could then process these data to make treatment recommendations or assist in the design of personalized medical devices or instruments [20]. Furthermore, in the field of personalized medicine, ML can enhance and optimize the performance of biomedical devices, particularly imaging instruments. Ultimately, ML has the potential to serve as the backbone that connects the dots between data-driven insights, cutting-edge technology, and the overarching goal of healthcare that revolves around the individual patient, ultimately shaping the future of medicine into a more personalized, precise, and effective endeavor.

**Figure 2:**
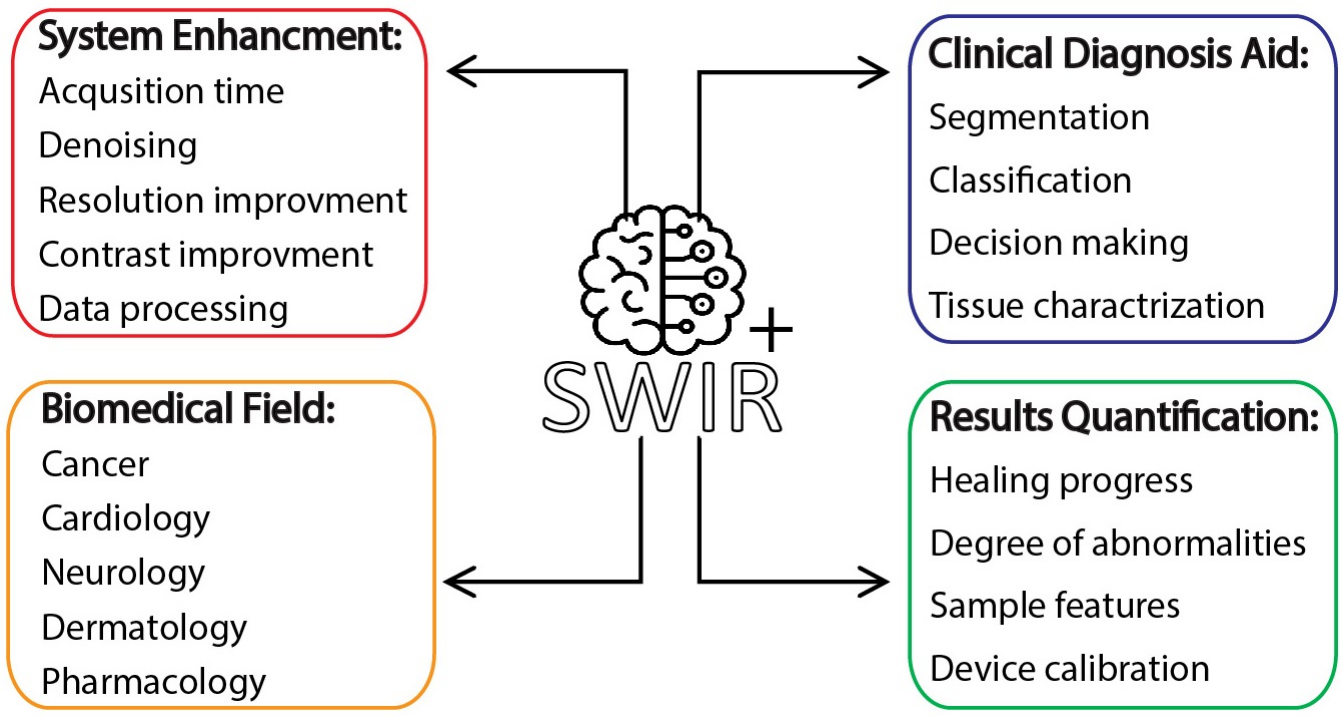
Categoriesof ML-powered SWIR techniques in the biomedical field.

This review aims to delve into diverse applications of ML-empowered SWIR techniques in the domains of personalized medicine and the visionary concept of individualized bioinstruments, where these models can undertake invaluable tasks, such as aiding in clinical diagnosis and result interpretation, optimizing system efficiency, and facilitating quantitative measurements. This review is motivated by the transformative impact of personalized medicine in healthcare, aiming to tailor interventions based on individual genetic, molecular, and clinical profiles. Focusing on SWIR techniques in biomedical optics, the research explores SWIR’s potential to enhance diagnostics, imaging, and therapeutic interventions by penetrating biological tissues with reduced attenuation. By integrating SWIR with machine learning, the study seeks unprecedented accuracy in disease detection and treatment guidance. Despite promising biomedical demonstrations, the clinical potential of SWIR remains underexplored. This paper demonstrates how the synergy between SWIR imaging and ML is reshaping biomedical research. The emphasis is placed on scrutinizing studies involving three prevalent SWIR techniques in preclinical and clinical settings, namely: fluorescence imaging, multi/hyperspectral imaging, and optical coherence tomography (OCT) systems. Figure 2 illustrates the areas in which ML can assist SWIR techniques in the field of biomedical research. In Section 2, the paper details the principles of SWIR modalities, providing a comprehensive understanding of these advanced imaging techniques. Section 3 introduces ML principles, outlining fundamental concepts, followed by an overview of common ML paradigms and model types. In Section 4, a thorough review explores studies that combine ML models with SWIR techniques. Section 5 discusses challenges, limitations, and future directions, providing insights into the evolving landscape of SWIR and ML integration in biomedical research. Finally, Section 6 provides the conclusion of the study.

## 2. Common SWIR Imaging Technologies

### 2.1. Fluorescent Imaging

Fluorescent (or fluorescence) imaging is a commonly used technique in the field of biomedicine, employed to image samples ranging from the cellular level on a microscope slide to larger tissue samples in vivo [21]. In clinical settings, fluorescence imaging is now employed as a tool for intraoperative guidance during surgeries in various configurations suitable for both open and laparoscopic surgery with potential applications, including tumor delineation, metastasis detection, and nerve visualization multiplexing [22]. Biomedical images generated in the SWIR frequency range offer striking contrast, precisely outlining tumor boundaries. Surgeons utilize these images to guide intricate procedures, ensuring accurate tumor removal while minimizing impact on healthy tissues. These compelling images not only demonstrate the immediate clinical relevance of SWIR fluorescence in enhancing surgical precision, but also emphasize its potential to revolutionize intraoperative decision making. This application captures the large interest of readers by highlighting the tangible benefits of SWIR fluorescence imaging in improving surgical outcomes and paving the way for more effective and targeted interventions in the field of personalized medicine. Fluorescent imaging has also been applied to pharmacology, offering real-time monitoring of drug distribution within the vasculature of animals [23].

**Figure 3:**
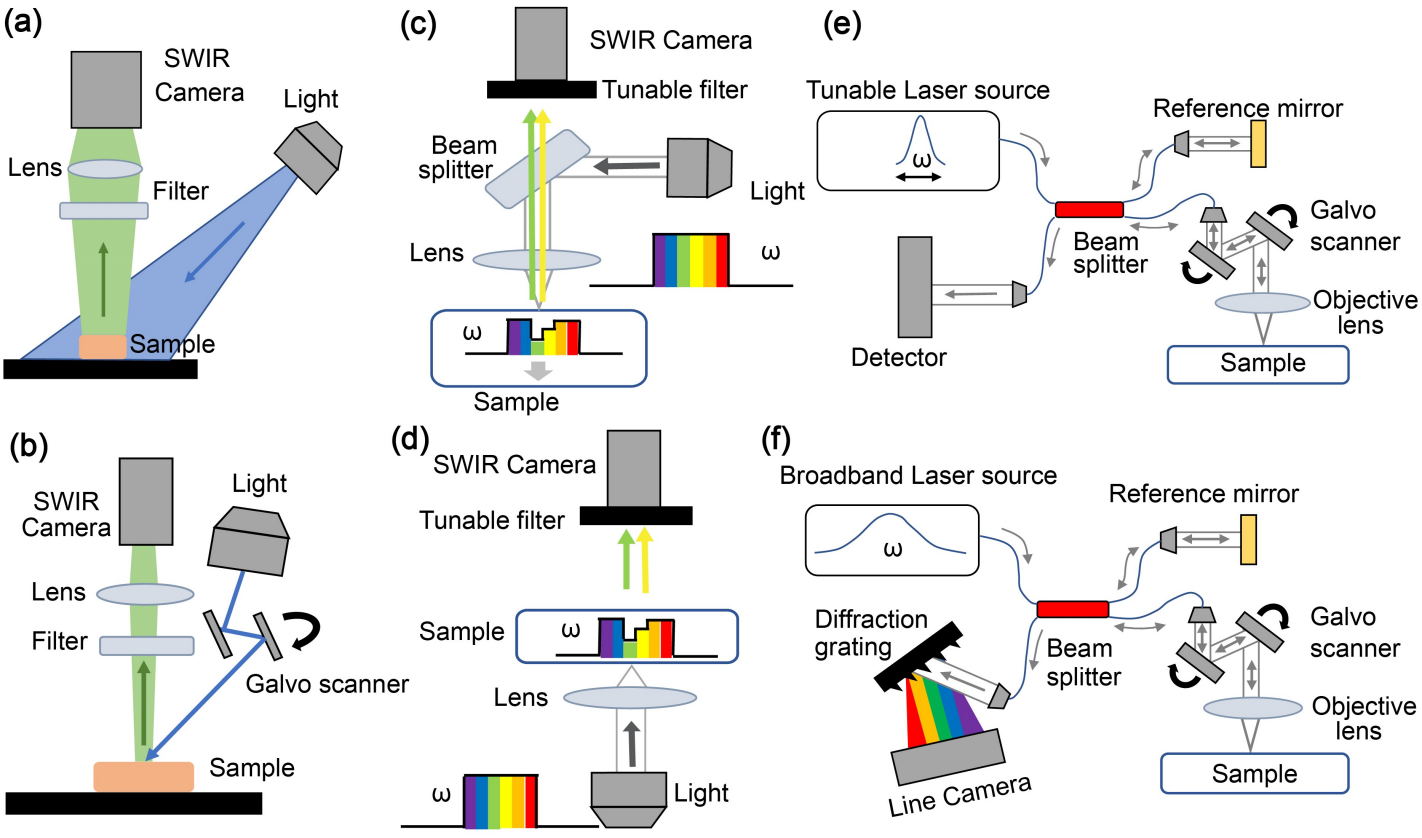
Schematic of SWIR techniques. (**a**) SWIRF system with wide-field epi-illumination method, (**b**) SWIRF with FMT configuration; hyper/multi spectral imaging with (**c**) reflectance and (**d**) transmittance setting; OCT systems with (**e**) swept source and (**f**) spectral domain configuration.

In this method, fluorophores within the sample are illuminated by light at the excitation wavelength, and the resulting emitted fluorescent light is captured by an SWIR camera [21]. Figure 3a illustrates the schematic of the epi-illumination SWIR fluorescent (SWIRF) imaging technique. A common approach for illuminating the sample is the wide-field configuration, where the entire sample is illuminated simultaneously, see Figure 3a [21,24]. However, as the emitted light from different points within the sample interacts, the signals received by the camera are semi-quantitative. For quantitative results, the fluorescence molecular tomography (FMT) method is recommended (Figure 3b), involving the raster scanning of the sample surface with a narrow light beam [21,24]. FMT, subsequently, employs an inverse modeling approach to generate a three-dimensional tomogram depicting the distribution of fluorophores within the sample. One key downside of FMT, however, is the relatively long acquisition and data processing times.

### 2.2. Multispectral/Hyperspectral Imaging

Spectroscopy is a measurement approach to characterize biological tissues by interrogating their responses to a spectrum of light or collection of wavelengths [25]. When a sample is imaged continuously across a range of wavelengths, the technique is referred to as hyperspectral imaging. Conversely, if the sample is imaged within discontinuous wavelength intervals, it is called multispectral imaging. Both approaches can yield spectra of the sample in either a reflectance (Figure 3c) or transmittance configuration (Figure 3d). Analyzing the recorded spectrum of each camera pixel allows for getting insight into the chemical makeup of the given part of the tissue [25,26].

In the SWIR range, spectroscopy reveals more distinct features in the spectra of tissue constituents, such as water, lipid, and collagen, compared to shorter wavelengths [4,27]. Furthermore, incident light in the SWIR range is less affected by scattering events, enabling deeper exploration of tissue surfaces for targeted molecules [25]. These advantages of SWIR spectroscopy offers heightened sensitivity to tissue constituents, facilitating enhanced characterization of variations in tissue composition concentrations resulting from conditions such as atherosclerotic plaque, tumors, and skin burns [4,26]. Spectroscopic systems hold the potential to serve as a virtual method for histology, where excised tissues are immediately analyzed by intraoperative post-biopsy procedures without the necessity of staining with chemical agents. Additionally, to study brain function in the field of neuroscience, hyperspectral images generated in the SWIR frequency range provide intricate details about oxygenation levels and metabolic activities in different brain regions, offering a comprehensive view of neural processes. These captivating images not only contribute to our understanding of complex brain functions, but also hold promise for unraveling mysteries related to neurodegenerative disorders. It is worth mentioning that one limitation of spectroscopic methods is their inherent lack of depth resolution, which means they provide a broad overview of the chemical composition of tissue beneath the surface without precise discrimination of information at specific depths.

### 2.3. Optical Coherence Tomography (OCT)

OCT is a method of interferometry that creates high-resolution 2D and 3D images of biological tissues at the micron level [28]. A prevalent type of OCT is Fourier-domain OCT, available in two configurations: swept source (Figure 3e) and spectral domain (Figure 3f). In both configurations, elastically back-reflected light from various tissue structures interfere with a reference light beam, generating a fringe pattern in the Fourier domain [28]. Applying Fourier analysis on this pattern transforms the “wavenumber” space (k-space) information into the “physical length” space (z-space). The absolute values of the complex numbers resulting from this Fourier transformation constitute the structural image of the tissue. In addition, the phase of the Fourier transformation result represents the differences in optical path lengths between tissue layers and the reference mirror. OCT functional modalities tied to phase, such as photothermal (PT)-OCT [29] and optical coherence elastography (OCE) [30], leverage phase variations over time to obtain additional information about tissue molecular and mechanical characteristics. In such modalities, the supplementary phase information automatically overlays on the high-resolution structural OCT amplitude images, creating a depth-resolved map for the additional properties of the tissue.

OCT and its extensions find diverse applications in both preclinical and clinical realms, spanning ophthalmology, cardiology, dermatology, oncology, and beyond [28]. By far, the largest clinical impact and area of application of OCT is in ophthalmology that traditionally utilized NIR light in the 800–870 nm range. More recently, however, ophthalmic OCT systems take advantage of reduced scattering at longer wavelengths and employ SWIR wavelengths of 1050 nm to enhance imaging depth and image quality, specifically for the comprehensive visualization of deeper regions of the posterior eye. OCT images generated in the SWIR frequency range offer unprecedented clarity, capturing detailed cross-sectional views of retinal structures. These captivating images not only showcase the potential of SWIR-based OCT in early detection and monitoring of eye conditions, such as macular degeneration and diabetic retinopathy, but also emphasize its role in advancing our understanding of ocular health. By highlighting the immediate clinical impact of SWIR-based OCT on visualizing intricate retinal details, this application captures the large interest of readers, underlining its potential to revolutionize ophthalmic diagnostics and contribute to the broader landscape of biomedical imaging. In other areas of application of OCT (e.g., cardiology), these systems typically incorporate SWIR light sources operating at 1310 ± 90 nm, to effectively mitigate light attenuation caused by scattering. This extension of the optical spectrum allows light to penetrate biological tissues to a depth of 2 to 4 mm, offering promising prospects for advanced medical imaging [28]. In cardiology, intravascular OCT (IV-OCT) systems, designed in a catheter-based configuration, scan arteries for detailed analysis of plaque characteristics in coronary disease and even to visualize the location of struts used in treatment [31]. OCT is also coupled with fluorescence [32,33] and spectroscopy techniques [34] to unveil deeper insights into biological tissues. OCT outcomes are quantifiable and contribute to the measurement of tissue scattering properties [35].

## 3. Machine Learning (ML)

ML is a subset of artificial intelligence (AI) that encompasses various methods of leveraging data to enhance machine performance through learning strategies [36,37]. In essence, ML automates the extraction of relevant features from historical data [38]. ML techniques have demonstrated diverse applications in both preclinical and clinical stages within the biomedical field [39,40,41]. ML units possess the capacity to aid clinicians in streamlining diagnoses by refining the accuracy and precision in decision making through pattern extraction and analysis of patient outcomes [42]. There are many other applications of ML in the biomedical field and the number of these applications is growing exponentially.

There are four primary paradigms of ML based on the nature of the dataset and learning objective: supervised, unsupervised, semi-supervised, and reinforcement learning [43]. In supervised learning, machines are trained using labeled datasets to map inputs to outputs, predicting classes (classification) or numerical values (regression). In unsupervised learning, the goal is to uncover patterns and similarities within unlabeled and unsorted datasets to categorize them (clustering) or to simplify the data while retaining as much relevant information as possible (dimensionality reduction). Semi-supervised learning pertains to scenarios where a small fraction of the training dataset is labeled, while the majority remains unlabeled. This approach bridges the gap between supervised and unsupervised algorithms, efficiently utilizing all available data. Reinforcement learning involves a ML unit learning through iterative exploration of its environment, maximizing cumulative rewards. This field is applicable to medical decision making, dynamic treatment regimes, and disease diagnosis.

Implementation of these learning paradigms involves developing an ML model and training the model on a portion of the dataset. The trained model should then exhibit accurate prediction on additional, previously unseen data, drawn from the same distribution as the training dataset (referred to as generalization) [44,45]. The field of ML offers a plethora of proposed models and algorithms to address a broad spectrum of challenges. Here, we highlight pivotal algorithms commonly applied in biomedical and SWIR imaging applications.

### 3.1. Advanced ML Methods

#### 3.1.1. Artificial Neural Network (ANN)

**Figure 4:**
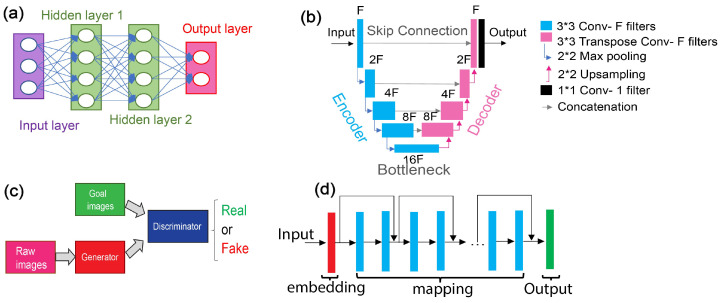
Illustration of the architectures of (**a**) a fully connected ANN, (**b**) U-Net, (**c**) GAN, and (**d**) Res-net.

The artificial neural network (ANN) model, drawing inspiration from the structure and information processing of biological brains, comprises nodes (i.e., artificial neurons) organized into layers (Figure 4a). Within each layer, a neuron performs a simple mathematical operation on its input signal and feed forwards the output to neurons in the subsequent layer [46]. Each neuron has a multiplication factor (also known as a weight) that is adjusted during the training process. These weights are adjusted through an optimization procedure to minimize the error of a loss function between the network’s output and the desired output (e.g., labeled outputs in supervised learning or energy function in unsupervised learning).

A key challenge in training an ANN lies in determining the optimal number of times the training data is passed through the model to adjust model parameters (i.e., the number of epochs) [46]. Overfitting occurs when an ANN captures unwanted data details (such as noise and experimental errors), leading to diminished performance. Conversely, stopping training prematurely can result in underfitting, where the ANN fails to comprehend the training dataset or generalize to new data. A recommended strategy involves selecting specific training parameters (e.g., epoch number, batch size, and learning rate) as hyperparameters and fine-tuning them through a grid search with various values. This strategy, combined with k-fold cross-validation, provides a less biased and more realistic evaluation of training.

It is essential to note that efficient training of an ANN requires a proper extracting of the most relevant features from the dataset [47]. The approach to feature selection differs between supervised and unsupervised problems. Performance of an ANN, particularly of regression models, degrades when the training dataset includes non-informative features.

#### 3.1.2. Auto Features Engineering Approach with Deep Neural Network (DNN)

Deep learning, a subset of ML, has redefined the landscape of AI by enabling models to automatically learn intricate patterns and representations from raw data. Unlike traditional feature selection methods that aim to manually extract relevant input attributes, deep learning models are designed to autonomously discern significant features through successive layers of abstraction [48]. Although feature selection is bypassed in deep learning, preprocessing steps such as normalization often enhance the dataset’s performance, resulting in improved DNN outcomes. While there is not a strict definition for the scale and size of a DNN, any ANN with more than one hidden layer holds potential for being considered a DNN. Technically, the number of DNN’s layers should be large enough that allows efficient backpropagation across layers during training for learning deeper features of the dataset. Yet, as DNN size increases, the number of parameters and weights grows, demanding a larger training dataset [49].

Creating a DNN involves employing different types of layers in the ANN architecture. Common layer types include fully connected (dense), convolutional, dropout, pooling, recurrent, and normalization layers [48]. The selection of these layers’ hinges on the model type (e.g., encoder–decoder), application (e.g., object detection, classification, and denoising), and dataset characteristics (e.g., videos, 2D images, 1D signals, and text). As such, numerous innovative architectural designs have been proposed to date that have revolutionized various domains, enabling more efficient and effective solutions (see Table 1).

The U-Net architecture, recognized for its success in biomedical image segmentation tasks, presents a unique architecture that features a contracting and expansive path, culminating in a U-shaped network (Figure 4b) [79]. This design enables U-Net to effectively capture both local and global contexts, making it particularly adept at tasks involving pixel-wise classification. On another front, Generative Adversarial Networks (GANs; Figure 4c) introduce a pioneering concept of adversarial training, pitting a generator and discriminator against each other to create realistic content [48]. GANs have found applications in image reconstruction, denoising, and data augmentation, offering a powerful framework for creative synthesis. Meanwhile, Residual Networks (ResNet) address the challenge of vanishing gradients in DNN by introducing residual connections that facilitate training of substantially deeper architectures (Figure 4d) [80]. This architecture, featuring residual blocks, has remarkably deep variants and has demonstrated superior performance across complex tasks such as disease prediction and image registration/fusion, showcasing its potential to unravel complex patterns within data.

### 3.2. Conventional ML Methods (Features Engineered Approaches)

#### 3.2.1. Support Vector Machine

**Figure 5:**
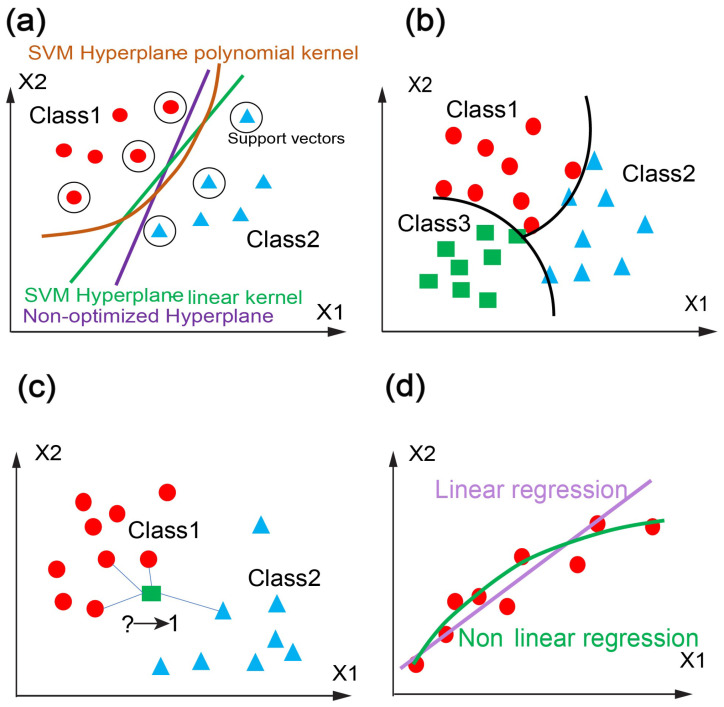
Illustration of prediction on dataset with ML models, (**a**) SVM, (**b**) Naive-Bayes classifier, (**c**) KNN, and (**d**) regression.

Support vector machines (SVMs) are a type of supervised learning models that operate within statistical learning frameworks, serving as robust prediction tools for classification tasks [81]. In SVM problems, an optimized N-1 dimensional hyperplane is identified to effectively classify datasets characterized by N selected features (Figure 5a). This hyperplane is drawn by using datapoints from each class that are situated closest to the other class, often referred to as support vectors. In the training phase, the primary objective is to maximize the margins, which is the distance between the hyperplane and the support vectors of each class. SVM algorithms employ a set of mathematical functions known as kernel functions to determine the optimized hyperplane. These kernel functions encompass various types, such as polynomial, radial basis function (RBF), and sigmoid. The choice of the kernel function should align with the distribution of the data.

#### 3.2.2. Naive-Bayes Classifier

Naive-Bayes classifiers refer to a set of classification algorithms that rely on Bayes’ Theorem [81]. This theorem enables the modeling of conditional probabilities of quantified statistical parameters. In these classifiers, the idea is to construct a probabilistic model of a class given selected features (Figure 5b). The shared assumption among these classifiers is that each pair of features in a dataset is independent of each other. Despite their simplicity, these supervised classifiers offer practical utility by effectively reducing the errors in misclassification.

#### 3.2.3. K-Nearest Neighbors

The K-nearest neighbors (KNN) algorithm stands as a non-parametric supervised ML model that predicts the grouping of an individual data point based on its proximity to other points [81]. While this algorithm can be employed in regression problems, its primary use lies in classification tasks. This approach involves assigning a label to a query data point (for classification) or calculating a continuous target value (for regression) considering the most similar neighbor points (the k nearest). In classification scenarios, the label of a given data point is determined through majority voting (Figure 5c). Although the implementation of this method is relatively straightforward compared to other ML models, the algorithm’s speed considerably decreases when dealing with large datasets.

#### 3.2.4. Regression

Regression refers to a class of supervised ML models that predict continuous values by identifying the optimal-fitting curve between input and output data (Figure 5d) [81]. Typically, in regression models, the objective is to minimize the mean squared error (MSE) between the fitted curve and the data points. Various regression methods include linear, polynomial, logistic, LASSO, and ridge methods. Regression proves to be a valuable tool for quantifying outcomes, particularly for biomedical applications. A notable challenge in regression lies in extrapolation for values beyond the range covered by the training dataset.

These models find diverse applications, notably in predicting disease progression, drug responses, and patient outcomes. For instance, in neurodegenerative diseases, regression models leverage biomarkers and clinical data to estimate the rate of disease advancement, guiding personalized treatment strategies. Additionally, they contribute to the realm of personalized medicine by predicting individual responses to specific drugs based on genetic and molecular data. Furthermore, regression models serve as valuable tools in experimental biology, aiding in the estimation of biological parameters and facilitating a deeper understanding of intricate cellular processes. Whether predicting survival rates or aiding in the development of diagnostic tools, the versatility of regression ML models underscores their significance in advancing biomedical research, fostering more precise clinical decision making, and ultimately contributing to improved patient outcomes.

## 4. Biomedical Applications of ML-Assisted SWIR Techniques

Table 1 summarizes the ML models and research highlights for a collection of recent SWIR work reviewed in this article. In general, ML can provide valuable support to SWIR techniques in three distinct ways: (1) Assistance in Diagnosis: ML can effectively analyze SWIR data and images to aid clinicians in making more accurate diagnoses; (2) Quantitative imaging and prognosis: ML can be leveraged to extract quantitative data from multifactorial SWIR datasets, enabling the extraction of precise information from biological systems (e.g., for disease staging); (3) Overcoming technological limitations: ML also has the potential to enhance the performance of SWIR technologies (e.g., increase imaging speed or image quality) without any hardware modification.

### 4.1. Assistance in Diagnosis

SWIR techniques offer promising solutions for biomarker detection in various diseases, particularly in cardiovascular diseases (CVDs) and cancers, which are leading causes of global hospitalization and mortality. ML as a transformative technology to analyze complex clinical data can assist in the interpretation of results from SWIR techniques, thereby enhancing diagnostic precision [82]. Remarkably positive outcomes have emerged from ML’s application in diagnosing both CVDs [83] and cancers [84], spanning various clinical stages. The text below scrutinizes recent advancements in ML-assisted diagnostics of SWIR images based on a clinical area of application.

#### 4.1.1. Cardiovascular Diseases (CVDs)

According to the World Health Organization (WHO), CVDs, a group of disorders related to the heart and its blood vessels, are responsible for an estimated 17.9 million deaths annually [85]. Atherosclerosis, characterized by the accumulation of substances such as lipids within the artery walls, contributes to a majority of CVDs [86]. In developed stages, atherosclerosis plaque leads to myocardial infarction, commonly known as a heart attack, by obstructing blood flow in the coronary arteries. Studies emphasize the critical role of plaque structure and composition, particularly its fibrous cap thickness as a determinant of high-risk plaques [87]. For example, thin cap fibroatheroma (TCFA), a primary type of high-risk plaque, typically exhibits a fibrous cap thickness of less than 65 µm [87]. These structural and molecular plaque instability hallmarks, however, are complex and frequently not detectable from angiographic images. IV-OCT serves as a diagnostic SWIR technique for various subclinical vascular diseases (SVDs) by enabling visualization of abnormalities within cardiovascular tissues during cardiac catheterization. IV-OCT excel at detecting structural abnormalities such as atherosclerotic plaque by providing micron-resolution cross-sectional images of the arterial walls [31].

**Figure 6:**
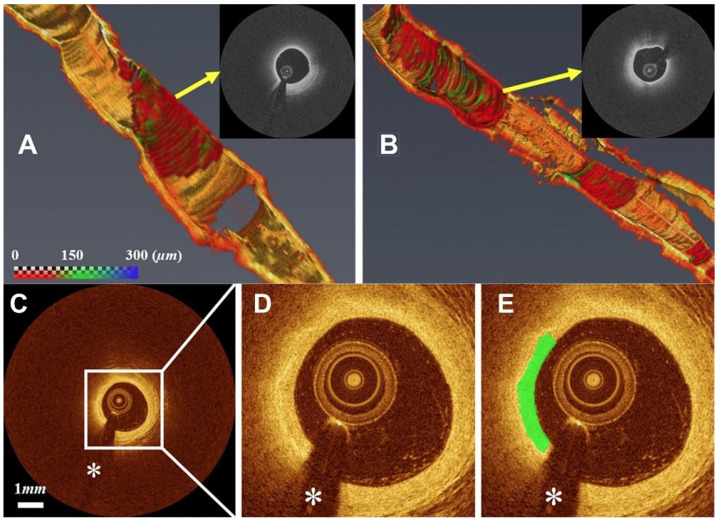
Automated detection results for TCFA from IVOCT pullbacks with ML. (**A**) Short lesion with TCFA, (**B**) long lesion with TCFA. The color bar in panel (**A**) represent the cap thickness. These three-dimensional (3D) visualizations results come from real-time analysis of each (**C**) captured cross sections by the ML model. For better visualization, panel (**D**) represents the zoomed view of the cross section and panel (**E**) indicates the zoomed view overlaid with a fibrous cap with a green color. The white asterisk (∗) is the guidewire shadow. Figures adapted with permission from [57].

However, classification of the degree of vulnerability of plaque to rupture from IV-OCT images is frequently inaccurate due to the multifactorial nature of light-matter interactions. As such, ML models, particularly DNN, have been widely employed to automatically characterize and classify both cardiac tissue [59,60] and plaque from IV-OCT results [56]. These studies leverage deep learning models to robustly identify various intracoronary pathological formations from OCT images. They showcase high accuracy, sensitivity, and specificity of the deep learning models, with values as high as 0.99 ± 0.01. These advancements hold substantial promise for improving the efficiency and accuracy of CVD diagnoses, contributing to enhanced patient care and outcomes in the field of pediatric cardiology. Furthermore, supervised DNN models were used in various studies to detect, classify, and segment TCFA from IV-OCT results (Figure 6) [57,58]. As an illustration, the automatic detection of TCFA from each B-scan through an IV-OCT pullback is rendered in a 3D map, with the cap thickness information depicted in Figure 6. In cases where supervised training with extensive labeled datasets is unfeasible, weak supervised learning methods have been proposed for TCFA detection [88]. These studies achieved high sensitivities and specificities for plaque classification, and demonstrated excellent reproducibility and efficient clinical analyses. These advancements have the potential to enhance patient care and streamline clinical workflows.

In the treatment stage, a recent study assessed the effectiveness of statin-based treatments on TCFA [89]. Using ML models, the study analyzed IV-OCT results from 69 patients over 8–10 weeks to predict changes in cap thickness. When dealing with limited datasets for ML unit training, alternative ML models can be utilized. For instance, a decision-tree-based model was trained using voxels from 300 images to classify plaques [61]. Identification of stents in IV-OCT scans aids cardiologists in assessing stent deployment and tissue coverage post-implantation. Diverse ML models, including DNN [66], Bayesian networks [65], decision trees [64], and SVM [63], have been employed to detect stents in IV-OCT results. A software tool called OCTOPUS V.1, incorporating various ML models, has been developed for offline analysis of both plaques and stents [62].

Furthermore, SWIR techniques have proven valuable for analyzing Heart Failure (HF), another prevalent form of CVD, affecting over 64 million people worldwide [76]. HF is characterized by the heart’s inability to efficiently fill with or pump blood due to structural or functional abnormalities. ML models have been utilized to enhance the quantification of edema; a key manifestation of HF [76]. Edema arises when an excessive volume of fluid accumulates in organs due to reduced blood pumping. Molecular chemical imaging in SWIR, as a hyperspectral technique, has been performed on patients. Spectral data obtained from these analyses were processed using Partial Least Squares (PLS) ML models to: (1) differentiate spectra from healthy and HF cases and (2) quantify the degree of edema.

#### 4.1.2. Cancer Diagnosis and Surgical Interventions

Cancer stands as the second leading cause of death globally, accounting for nearly 10 million deaths in 2020 [90]. Surgical procedures have been employed in 45% of cases for cancer treatment [72]. Precise intraoperative tumor delineation plays a pivotal role in enhancing the effectiveness of surgical interventions. Extension of fluorescent surgery in SWIR holds promising potential to aid clinicians in achieving more precise intraoperative tumor delineation. Within neurological surgery, specifically addressing gliomas—a prevalent malignant primary nervous system tumor—conventional practices rely on surgeons’ visual assessments aided by intraoperative histopathological findings from dissected tissues [50]. However, this approach lengthens procedure times and adds complexity and uncertainty. By leveraging deep learning models to analyze SWIRF data from collected tissue, real-time and highly accurate detection of tumor regions becomes achievable. In the study, employing a DNN on SWIRF data demonstrated the capacity to detect non-trivial features in images, yielding superior performance in identifying tumor regions (93.8%) compared to neurosurgeon evaluations (82.0%, *p* < 0.001) [50].

It is important to note that due to the multifactorial nature of SWIRF signals, relying solely on pixel intensity for tumor delineation can lead to errors. To address this challenge, a study pursued tumor delineation within small animals in vivo utilizing a multispectral SWIR approach [72]. To enhance decision accuracy, captured tumor region spectra were subjected to analysis by seven distinct ML models. Results revealed that despite subtle differences between tumor and non-tumor spectra, employing KNN models increased classification accuracy to 97.1% for tumors, 93.5% for non-tumors, and 99.2% for background regions. Another study focused on distinguishing malignant kidney tissues from normal tissues in an ex vivo setting [77]. Raman spectroscopy in the SWIR range was used to obtain spectra containing various peaks, reflecting tissue conditions (normal or malignant). To classify these spectra, a Bayesian ML model called sparse multinomial logistic regression (SMLR) was applied. The study showcased a classification accuracy of 92.5%, sensitivity of 95.8%, and specificity of 88.8% using this ML model.

**Figure 7:**
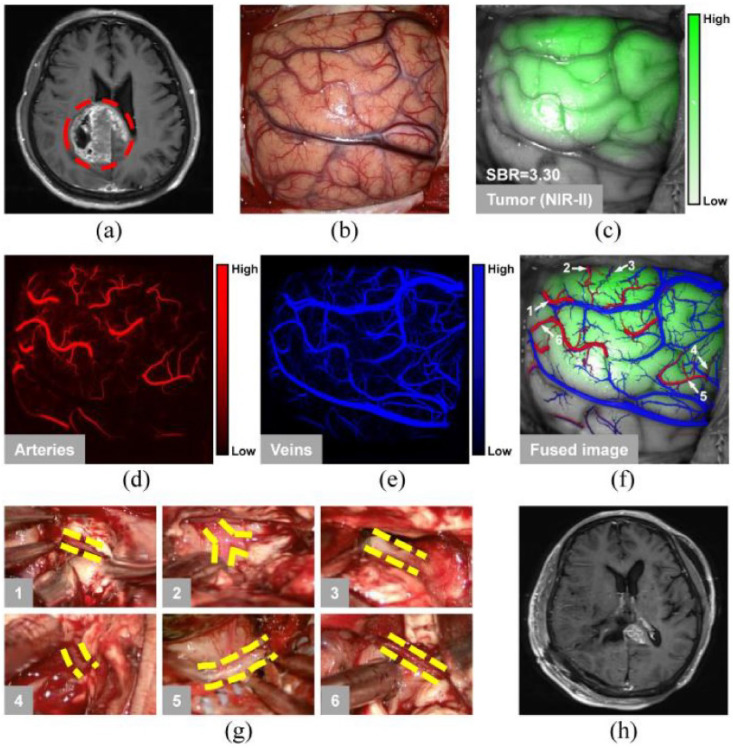
Showcase of blocking feeding arteries and tumor resection of a patient with GBM using the image-guided method. (**a**) MRI scans before surgery indicated a lesion in the right parietal (red circle). (**b**) Scan results with visible light after dural opening. (**c**) Scan of tumor with SWIRF method. NIR-IIb image of the (**d**) feeding arteries and (**e**) veins. (**f**) Integrated image of these separate scans. White arrows 1–6 correspond to the location of the tumor-feeding arteries. (**g**) Yellow lines show the feeding arteries blocked as a result of tumor resection during surgery. Panel 1–6 correspond to the vessels labeled as 1–6 in (**f**). (**h**) MRI scan shows the tumor was resected to the maximum extent after this image-guided surgery. Figure adapted with permission from [55].

Furthermore, as a pioneering advancement in the field of intraoperative surgical procedures for glioma resection in humans, a groundbreaking method has been devised leveraging multispectral fluorescence spanning the NIR and SWIR spectra (see Figure 7) [55]. The inherent high contrast images provided by SWIR technology empower surgeons to identify capillaries with unprecedented precision, detecting vessels as small as 182 μm, while enhancing the certainty of isolating tumor-feeding arteries. In order to process these images in real time, a DNN equipped with a U-Net structure was harnessed for the purpose of segmenting the intricate blood vasculature. This innovative approach not only revolutionizes intraoperative procedures but also underscores the potential of advanced imaging technologies to redefine the landscape of surgical interventions.

In surgical procedures, the accurate segmentation of vessels, overlapping structures, and junctions within SWIRF images emerges as a critical necessity. A previous study has demonstrated the application of DNN for vasculature segmentation purposes, in preclinical phases as a proof of concept [52]. The Iter-Net model, originally devised for retinal segmentation, accurately delineated murine vasculature captured in vivo using SWIRF imaging. Remarkably, the DNN not only achieved effective segmentation, but also collected supplementary vascular insights encompassing morphological details, discernment of vessel types (veins or arteries), and characterization of hemodynamic attributes. These accomplishments hold the potential to extend into the SWIRF-guided surgery, offering valuable assistance to surgeons during operative interventions.

An innovative method was reported for detecting tumoral epithelial lesions through the synergy of hyperspectral imaging and deep learning. Detecting these lesions early is crucial for effective cancer diagnosis and treatment planning. DNN RetinaNet was then utilized to automatically identify and classify these lesions. The findings highlight the effectiveness of this approach, offering a promising non-invasive solution for early-stage cancer detection, with potential implications for enhancing patient care and treatment strategies [91].

OCT and its extended modalities have gained prominence in cancer-related applications, due to the fine resolution, high acquisition rate, and millimeter-scale imaging depth, especially in intraoperative tumor margin evaluation. Moreover, compatibility of OCT with fiber optic technology enables miniaturization of its imaging head into diverse portable formats, such as handheld, needle probes, and single fibers, which enables precise assessment of tumor margins during breast-conserving surgeries [28]. By harnessing DNN, clinical OCT datasets sourced from patients with breast tumors have achieved remarkable outcomes, indicating a 98.9% accuracy in the classification of 44 normal and 44 malignant cases [92]. Additionally, the fusion of ultrahigh-resolution OCT systems operating in NIR and SWIR ranges, coupled with the assistance of a Relevance Vector Machine ML model, demonstrated an automatic detection capability for invasive ductal carcinoma within ex vivo human breast tissue, achieving an overall accuracy of 84% [93]. Further demonstrating the efficacy of OCT, a study employing an SVM model to classify OCT images from cancerous tissues yielded exceptional metrics with sensitivity, specificity, and accuracy values of 91.56%, 93.86%, and 92.71%, respectively, [94]. In microscopy, the integration of OCT and Raman spectroscopy yielded a comprehensive set of morphological, intensity, and spectroscopic features for ML models aimed at classifying cancer cells in an in vitro setting. By subjecting the images to analysis using three distinct ML models, namely linear discriminant analysis, KNN, and decision tree, the study achieved an impressive classification accuracy of 85% for distinguishing among five different types of skin cells [95]. A recent advancement involves the application of polarization sensitive (PS)-OCT, as a modality of OCT to provide higher contrast for differentiation and classification of malignant tumors, fibro-adipose tissues, and stroma within human excised breast tissues [96]. Employing leave-one-site-out cross-validation, an SVM model was trained to categorize the captured images. The outcome yielded an 82.9% overall accuracy when compared against histopathological results, substantiating the potential of PS-OCT in offering reliable cancer diagnostic insights.

Beyond the aforementioned primary SWIR imaging techniques, novel imaging systems have emerged in this spectral range. For example, an innovative technique grounded in orthogonal polarization imaging (OPI) within the SWIR range enabled size measurements of lymph nodes in both animal and human samples [78]. Given that cancer cells metastasize through the vascular and lymphatic system, this study harnessed deep U-Net models for high-contrast, label-free image analysis to automatically segment lymph nodes. These advancements highlight the profound impact of ML-assisted SWIR techniques in enhancing cancer diagnosis and surgical interventions.

### 4.2. Quantitative Imaging and Prognosis

ML has also proven to be powerful in quantifying signals obtained through SWIR techniques, offering insights into health-related measurements. For instance, the quantification of water and lipid contents in tissues holds value for monitoring physiologic levels. To this end, an SWIR probe designed for scanning thin tissues, utilizing diffused light, was constructed using three LED light sources and four source-detector separations [73]. Employing a DNN, the percentage of water and lipid components were estimated by analyzing the received signals in the detectors. Training the DNN involved simulating various conditions through precise Monte Carlo simulations. Results on phantoms demonstrated accurate quantification of water (2.1 ± 1.1% error) and lipid (1.2 ± 1.5% error) components using this approach. Expanding this study to the meso-patterned imaging (MPI) modality facilitated monitoring important physiological processes in clinics, including edema, inflammation, and tumor lipid heterogeneity [75]. In this context, hyperspectral MPI results were subjected to analysis by an SVM model for identifying subcutaneous brown adipose tissue.

In another study utilizing spectroscopy, in vivo skin parameters were quantified by applying two ML models to spectral reflectance results captured by sensors spanning from 450 to 1800 nm [74]. Quantifying skin parameters holds immense importance in dermatology, benefiting cancer diagnostics, wound healing, drug delivery, and related applications such as skin aging. The study introduced a theoretical model correlating skin physiological parameters to back-reflected light spectra. SVM and KNN models were then applied in a reverse modeling approach to derive skin parameters from the back-reflected spectra. Results from 24 human cases showcased favorable agreement between the prediction results from this non-invasive method and ground truth data.

**Figure 8:**
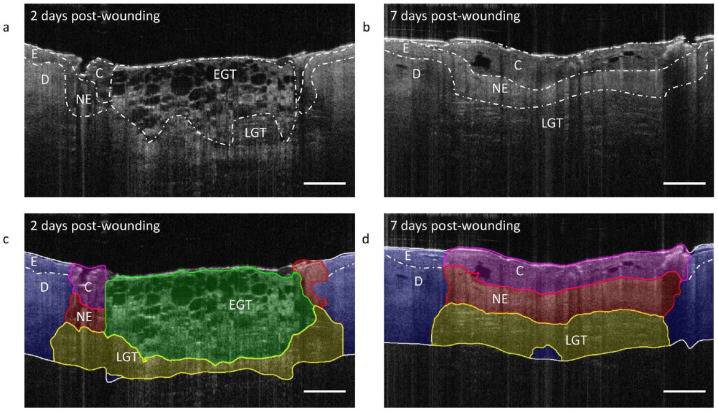
Monitoring results of wound healing. After (**a**) 2 and (**b**) 7 days, images acquired with OCT are shown with their segmented (**c**,**d**) regions in the corresponding OCT B-scan with ML model. The annotations are: blood clot (C); dermis (D); epidermis (E); early granulation tissue (EGT); late granulation tissue (LGT); neo-epidermis (NE). Scale bar = 500 μm. Figure adapted with permission from [69].

Within the domain of OCT, quantitative techniques have emerged, which are independent of the specific OCT system used for data acquisition for clinically relevant measurements. ML models have also proven to be capable in enhancing precision in quantification of OCT results. For instance, in cardiology, quantifying lipid content aids clinicians in determining the growth stage of atherosclerotic plaques. Beyond cap thickness, lipid-rich necrotic cores serve as significant indicators of high-risk plaques [97]. A study employed a discriminant analysis ML model on spectral and attenuation data, based on acquired OCT spectra to quantify key chemicals, such as lipid, collagen, and calcium, in phantoms and swine ex vivo tissue [98]. This advancement has significant implications for personalized medicine, as it enables precise depth localization of lipids and necrotic cores in coronary plaques, improving the interpretation of IV-OCT data and facilitating tailored treatment approaches. In the field of dermatology, a U-Net model was applied to OCT results from wounds to quantify wound morphology during the healing process [69]. This approach automatically detects wound morphology and quantifies volumetric transitions throughout treatment, promising a non-invasive and real-time method for wound monitoring (Figure 8).

In the field of ophthalmology, ML assists in measuring and quantifying biomarkers in different parts of the eye on OCT scans. A novel deep learning method, the residual U-Net, was introduced for the automated segmentation and quantification of choroidal thickness (CT) and vasculature [99]. Even with limited data, the precision achieved by this approach was comparable to that of manual segmentation conducted by experienced operators. High agreement is observed between manual and automatic segmentation methods, with intraclass correlation coefficients (ICC) exceeding 0.964 on 217 images. Furthermore, excellent reproducibility is demonstrated by the automatic method, with an ICC greater than 0.913. These results highlight the effectiveness of deep learning in accurately and consistently segmenting choroidal boundaries for the analysis of CT and vasculature. The impact of accurate choroidal segmentation using deep learning on personalized medicine concept is substantial, as it contributes to early disease detection, more precise diagnoses, better disease progression monitoring, and overall improvements in the efficiency of ocular healthcare.

In ophthalmology, understanding the human vitreous structure is vital, given its substantial age-related variations, but in vivo study limitations have persisted due to the vitreous transparency and mobility. Although OCT is routinely used to identify boundaries within the vitreous, the acquisition of high-resolution images suitable for generating 3D representations remains a challenge. A study used ML-based 3D modeling, employing a CNN network trained on manually labeled fluid areas [100]. The trained network automatically labeled vitreous fluid, generating 3D models and quantifying vitreous fluidic cavities. This innovative modeling system introduced novel imaging markers with the potential to advance our understanding of the aging processes and the diagnosis of various eye diseases, contributing significantly to ocular health assessment and clinical management.

For precise eye disorder quantification, particularly in complex cases with distorted anatomy, automated segmentation of fluid spaces in OCT imaging is crucial. A novel end-to-end ML approach was presented for combining a random forest classifier for accurate fluid detection and an efficient DeepLab algorithm for quantification and labeling [101]. The method achieves an average Dice score of 86.23%, compared to manual delineations by an expert. This approach promises to significantly improve automated fluid space segmentation and quantification in OCT imaging, enhancing clinical management and monitoring of eye disorders, particularly in complex cases.

Accurate quantification of intrachoroidal cavitations (ICCs) and their effect on visual function is paramount, particularly in high myopia. A study introduced a new 3D volume parameter for ICCs, addressing the need for precise quantification [102]. A significant knowledge gap exists regarding the relationship between 3D ICC volume and visual field sensitivity, and this study quantifies this correlation. Through deep learning-based noise reduction, the study quantified ICCs in 13 eyes with high myopia. It revealed negative correlations between ICC volume, length, depth, and visual field metrics, highlighting the role of quantification in understanding ICC impact. This research introduces a novel parameter for ICC assessment, enhancing our understanding of their effect on visual function. This has the potential to improve clinical detection and precise quantification of ICC pathology in high myopia, ultimately benefiting patient care and management.

Another success of ML models in OCT is enabling quantification of cellular microstructures that are finer than the inherent resolution of OCT. Alterations in light scattering patterns stemming from distributed particles within the phantoms yield significant optical signals. ML has proven to be successful in quantifying such non-trivial patterns in the OCT results. In a study, OCT images of assorted tissue-mimicking phantoms underwent analysis via SVM models to quantify speckle through texture [103]. Furthermore, a DNN was employed to accurately estimate fundamental parameters encompassing the count of scatterers within a resolution volume, lateral and axial resolution, as well as signal-to-noise ratio (SNR) by analyzing local speckle patterns within the OCT images [104]. These networks can also find utility in calibrating OCT systems for exceptionally precise measurements of the attenuation coefficient, exemplifying their potential to enhance the precision and versatility of OCT techniques.

Theoretical models developed for PT-OCT signals demonstrate that PT-OCT signals are influenced by multiple parameters, necessitating consideration of the interplay between them for accurate quantification of titers of molecules of interest [16,29]. Recent applications of SVM models to PT-OCT data successfully classified phantoms based on their lipid content irrespective of their depth within the sample [71]. This study can have a significant impact on personalized medicine by enhancing diagnostic capabilities and improving patient outcomes through a better understanding of lipid-related aspects of various diseases. OCE is another modality of OCT that can provide mechanical-related properties of tissues. A DNN was applied on obtained mechanical properties data from phantoms mimicking tissues with different mechanical elasticity to extract mechanical properties [105]. This advancement in elastic property estimation using DNN from OCE data holds the promise of enhancing personalized medicine by providing clinicians with real-time, non-invasive tools for assessing tissue characteristics and tailoring treatments to individual patient needs.

### 4.3. Overcoming Technological Limitations

**Figure 9:**
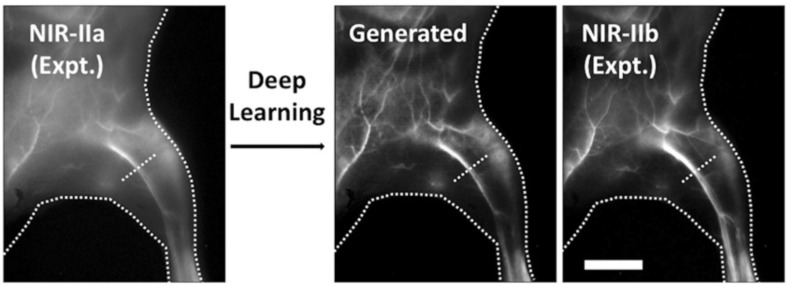
Generativemodel of NIR-IIb from NIR-IIa. Contrast enhancement of SWIRF by generating images with a GAN network to resemble a NIR-IIb image from a NIR-IIa input image (scale bar, 5 mm). Figure adapted with permission from [51].

ML offers a promising solution to address certain inherent limitations in SWIR techniques. A notable restriction of SWIRF imaging is the lack of FDA-approved dyes tailored for efficient emission in the SWIR band [8]. Currently, Indocyanine Green (ICG) stands as the lone FDA-approved option, primarily emitting in the NIR-I region (700–900 nm), albeit with a relatively weak emission tail in the SWIR spectrum [8]. A study aimed to harness the power of DNN to convert SWIRF images captured within the NIR-I/IIa window (900–1300 nm) using ICG dyes into comparable images captured in the NIR-IIb (1500–1700 nm) range with SWIR-specialized dyes (see Figure 9) [51]. Results showcased significant enhancement in the signal-to-base ratio (>100) for in vivo lymph node imaging, along with notable improvements in tumor-to-normal tissue ratios (>20) and tumor margin detection through imaging epidermal growth factor receptor after processing ICG images by the trained DNN.

Another challenge in fluorescent imaging pertains to accurately reconstructing a 3D map of fluorophore distribution, a task constrained by complicated inverse modeling problems. DNNs demonstrated capability to directly render the 3D distribution of fluorophores from raw data, eliminating the need for complex inverse modeling calculations [53]. Increasing the imaging depth in SWIRF has also become possible with DNN models. While the typical maximum penetration depth in soft tissues in the SWIR range spans from 4 to 6 mm, penetration depth decreases to 1.4 mm for brain imaging due to higher tissue scattering. To overcome this limitation, a DNN was trained using images acquired through a two-photon illumination technique. Reconstructing SWIRF images with this network resulted in enhanced SNR in deeper tissue layers [54]. This advancement facilitated 3D volume reconstruction of brain tissues with enhanced details without compromising temporal resolution.

ML models have also addressed some systemic limitations of OCT imaging, such as enhancing image contrast, extending the imaging range, and correcting for degradation of spatial resolution with depth. For instance, dual DNNs have directly enhanced axial resolution from raw interference fringe signals and subsequently reconstructed B-scans to reduce speckle noise [106]. Likewise, DNNs with GAN structures have been trained with different variations of the OCT system to produce speckle-free images [107,108]. For OCT retinal images, speckle noise was effectively removed using CNN [109] and GAN [110] deep networks that autonomously learned from training data, eliminating the need for manual parameter selection. OCT’s challenge of balancing lateral resolution and depth of focus was successfully met by a DNN, which reconstructed out-of-focus en-face images through a GAN structure [111]. ML models have also accelerated OCT systems by reducing required spectral datapoints, followed by DNN-based reconstruction to eliminate aliasing artifacts resulting from undersampling [112,113]. Additionally, to combat axial resolution degradation due to light dispersion in tissues, a modified U-Net architecture has proven effective in compensating for chromatic dispersion in OCT [67].

Beyond conventional OCT, ML models have helped overcome technological limitations of functional extensions of OCT. For instance, in OCT-Angiography (OCTA), the quality of results and the field of view have an inverse relationship. To address the issue, a DNN was employed to transform low-quality outcomes obtained from a 6 mm by 6 mm field of view into high-quality results acquired from a 3 mm by 3 mm field of view [114]. ML has also been used to improve the performance of OCT phase-tied modalities, such as PT-OCT. The quality of results in these modalities have a direct relation with the length of captured time trace OCT phase signal over each A-line. As such, these modalities typically suffer from a low acquisition rate, which renders them impractical for clinical use. In a study, using an ANN model, SNR of images acquired with PT-OCT using short acquisitions were improved to SNR values normally offered by very long acquisition times [70]. Another interesting potential of ML is in extracting additional information from OCT datasets that are not directly accessible via OCT raw images. In a relevant work, a DNN model employing a GAN architecture synthesized PS-OCT images from raw OCT intensity images, avoiding the need for additional hardware needed to construct a PS-OCT system [68].

Similar to OCT, ML has been shown to enhance performance in various other SWIR modalities. For example, DL classifiers were utilized to improve the detection accuracy of otoscopy for diagnosing middle ear effusions [115]. Middle ear effusions, commonly associated with ear infections, are a prevalent medical issue, particularly in pediatric patients. The traditional diagnostic process often involves invasive procedures, which can be uncomfortable and impractical, especially for children. Leveraging advanced DL models, the system analyzes SWIR images of the ear canal and tympanic membrane, identifying specific features indicative of effusion with a specificity and sensitivity over 90%.

## 5. Challenges and Perspectives

The advent of emerging technologies in SWIR has significantly advanced in vivo imaging quality in terms of resolution, imaging depth, and SNR. Furthermore, exploring biological tissue spectra within this wavelength range reveals distinct features of tissue constituents previously unseen in shorter wavelengths. The recent years have witnessed significant advancements in SWIR techniques, indicating a promising trajectory for the future of clinical applications as well as individualized bioinstruments. However, inherent challenges within SWIR imaging, encompassing limitations related to cost, performance, and the accessibility of SWIR light sources and detectors, cast a nuanced light on the broader application of SWIR techniques. The pursuit of novel materials to enhance cost-effectiveness and sensitivity in SWIR devices, along with addressing the scarcity of FDA-approved fluorophores tailored for SWIR emission, remains imperative. The complexity introduced by the limited availability of simulation and modeling tools adds another layer to the challenges faced in SWIR-based biophotonics research. Moreover, the relative scarcity of clinical studies utilizing SWIR, especially when compared to well-established modalities such as ultrasound imaging, underscores the need for expanded research efforts to unlock the full potential of SWIR in diverse clinical applications.

Another challenge is the limited number of clinical studies employing the SWIR technique, especially when compared to well-established modalities such as ultrasound imaging. In the future, an increase in clinical studies using SWIR could unlock opportunities for improved disease diagnosis and prognosis. For instance, associations have been identified between ocular health and various diseases, Parkinson’s disease, hypertension, CVDs, cerebrovascular disease, dyslipidemia, chronic kidney disease, and neurodegenerative disorders [116,117,118,119]. The eyes serve as a window into the body’s overall health, and emerging evidence suggests that ocular manifestations can provide valuable insights into the presence and progression of systemic diseases. Integrating OCT into comprehensive clinical assessments may offer a non-invasive means of diagnosing and monitoring systemic diseases, contributing to a more holistic approach in healthcare. For example, Parkinson’s disease, characterized by dopaminergic neuron degeneration, exhibits changes in the retina. OCT allows high-resolution imaging of retinal layers implicated in Parkinson’s pathology, potentially serving as an early indicator of the disease. Continued exploration and application of SWIR techniques, particularly in conjunction with OCT, could play a pivotal role in advancing our ability to diagnose, understand, and manage systemic diseases through ocular health indicators. Overcoming hardware design challenges for SWIR systems while miniaturizing bioinstruments for point-of-care applications remains essential.

Notwithstanding these challenges, the applications of SWIR technology are burgeoning in various biomedical domains, particularly clinical imaging, enabling the visualization of previously imperceptible details. The extraction of valuable insights from data captured through SWIR techniques greatly contributes to data-driven personalized medicine. Simultaneously, ML emerges as a disruptive technology, fortifying SWIR techniques in myriad ways, including object detection, segmentation, image reconstruction, result quantification, decision making, and setup performance enhancement. The future envisions an increasingly pronounced role for ML, particularly DNNs, in advanced and automated diagnostics. The rapid development of ML algorithms anticipates more advanced image analysis tools extracting valuable insights from medical images with unparalleled precision. This will enable healthcare providers to tailor treatment plans more precisely to each patient’s unique biology, leading to more effective and personalized therapeutic interventions.

Yet, challenges persist in integrating ML into clinical systems. A significant hurdle for data-driven ML models, such as DNNs, is their generalization capability, relying on the quality of the training dataset. The standardization and integration of data from various imaging modalities into a cohesive and interoperable framework to feed ML models remains a formidable task. Different healthcare institutions and imaging modalities often use diverse formats and standards for data storage, challenging the creation of unified datasets and ML models that work seamlessly across institutions. Compiling a comprehensive dataset from clinical data proves intricate due to a wide data distribution, result dependency on various machines, and ethical restrictions on accessing medical data. Biased training data can lead to biased ML models, causing disparities in diagnosis and treatment. Ensuring fairness and mitigating bias in training datasets is crucial in healthcare applications. Developing weakly supervised DNN models that require fewer labeled training data emerges as a plausible solution. Uptake of ML-assisted innovations by clinicians necessitates an emphasis on explainable AI approaches, including measures such as simplifying models, incorporating interpretability tools into solutions (e.g., attention maps or feature importance maps), leveraging referenceable data for training, and promoting human-in-the-loop decision making. The lack of robust criteria for recommending optimal settings and architectures for ML models, particularly for ANNs, underscores the need for a systematic approach in proposing optimized models based on given datasets.

In essence, the collaborative synergy between SWIR techniques and ML is poised to redefine the landscape of biomedical research and clinical applications. As these technologies continue to evolve, interdisciplinary efforts of researchers, engineers, and clinicians will be instrumental in realizing their full potential. The challenges outlined, while formidable, are surmountable through concerted research endeavors, opening the way for a future where SWIR and ML contribute seamlessly to the advancement of personalized medicine and transformative healthcare outcomes.

## 6. Conclusions

This review delved into the applications, challenges, and future trajectory of ML in SWIR techniques within the biomedical field, particularly in SWIRF, OCT, and multi/hyperspectral imaging. ML models have augmented diagnostic procedures, mitigated system limitations, and facilitated result quantification. In essence, the synergy between SWIR techniques and ML represents an exciting frontier with the potential to significantly advance biomedical applications. The intersection of these two domains promises to further revolutionize the field of medical imaging by unlocking new dimensions of precision and accuracy in diagnosis and treatment. As clinical applications for SWIR techniques continue to expand, the demand for cutting-edge ML models to assist clinicians in image analysis is poised to grow, heralding an era of unprecedented individualized bioinstrumentation that can tailor medical care to the unique needs of each patient. In this era, the fusion of SWIR techniques and ML will not only be transformative but also hold the promise of improving healthcare outcomes for individuals worldwide.

## Figures and Tables

**Table 1:** Examplesof applications of ML models in different SWIR modalities.

	Ref.	ML Model	Explanation
SWIRF	[50]	Deep CNN Network	Intraoperative glioma tumor detection
	[51]	Deep GAN Network	Overcoming limitation of fluorescent probe in SWIR by converting NIR-a to NIR-b results
	[52]	Deep Iter-Net	Segmentation on vasculature, in vivo
	[53]	Deep FCC Network	3D rendering on fluorescent results with the FMT method
	[54]	Deep Scale-recurrent Network	Image 3D reconstructing for increase SNR from deeper tissue layers
	[55]	Deep U-Net	Enhancing the precision of surgical resection of gliomas
OCT and its modalities	[56,57,58]	Deep Network	Automated coronary plaque classification for risk assessment
	[59,60]	Deep Network	Cardiac tissue characterization for detection of Kawasaki disease’s biomarkers
	[61]	Decision Tree	Coronary plaque classification with smaller dataset for training the ML model
	[62,63]	SVM	Automated stent coverage analysis and detection
	[64]	Decision Tree	Automatic stent detection from IV-OCT pullback results
	[65]	Bayesian Network	3D stent detection from IV-OCT results
	[66]	Deep Network	Stent detection under deep tissue coverage
	[67]	Deep U-Net	Automated dispersion compensation in OCT for improvement in axial resolution
	[68]	Deep GAN Network	Synthesize PS-OCT images from conventional OCT images to tackle the limitation of conventional OCT system in providing birefringence-related contrast
	[69]	Deep U-Net	Quantifying wound morphology as an automatic method to monitor wound healing
	[70]	Deep FCC Network	Enhancing imaging rate of functional phase-related OCT extensions
	[71]	SVM	Quantification of lipid content with OCT and its modalities on phantoms
Hyper/multi spectral	[72]	KNN	Enhancing intraoperative tumor delineation in mouse, in vivo
	[73]	Deep Network	Quantification of water and lipid in phantoms
	[74]	KNN and SVM	Measuring skin parameters on humans
	[75]	SVM	Quantitative label-free brown adipose tissue characterization
	[76]	Partial least squares	Quantitative description of edema description
	[77]	Bayesian classifier	Distinguishing malignant kidney tissue from normal tissues
	[78]	Deep U-Net	Lymph nodes segmentation and size measurement

## Data Availability

No data available.

## Abbreviations

The following abbreviations are used in this manuscript:

AI	Artificial Intelligence
ANN	Artificial Neural Network
CVD	Cardiovascular Disease
DNN	Deep Neural Network
EGT	Early Granulation Tissue
FMT	Fluorescence Molecular Tomography
GAN	Generative Adversarial Networks
HF	Heart Failure
InGaAs	Indium Gallium Arsenide
ICG	Indocyanine Green
IV	Intravascular
ITAR	International Traffic in Arms Regulations
KNN	K-Nearest Neighbors
LGT	Late Granulation Tissue
LP	Long Pass
ML	Machine Learning
MSE	Mean Squared Error
MPI	Meso-Patterned Imaging
NIR	Near Infrared
NE	Neo-Epidermis
OCE	Optical Coherence Elastography
OCT	Optical Coherence Tomography
OPI	Orthogonal Polarization Imaging
PLS	Partial Least Squares
PT	Photothermal
PS	Polarization Sensitive
RBF	Radial Basis Function
ResNet	Residual Networks
SWIR	Short-Wave Infrared
SWIRF	Short-Wave Infrared Fluorescent
SNR	Signal-to-Noise Ratio
SMLR	Sparse Multinomial Logistic Regression
SVD	Subclinical Vascular Disease
SVMs	Support Vector Machines
TCFA	Thin Cap Fibroatheroma
WHO	World Health Organization
